# ApoE4-associated phospholipid dysregulation contributes to development of Tau hyper-phosphorylation after traumatic brain injury

**DOI:** 10.1038/s41598-017-11654-7

**Published:** 2017-09-12

**Authors:** Jiqing Cao, Farida El Gaamouch, James S. Meabon, Kole D. Meeker, Li Zhu, Margaret B. Zhong, John Bendik, Gregory Elder, Ping Jing, Jiahong Xia, Wenjie Luo, David G. Cook, Dongming Cai

**Affiliations:** 1 James J. Peters VA Medical Center, Neurology Service, Bronx, NY 10468 USA; 2 The Central Hospital of Wuhan, Tongji Medical College, Huazhong University of Science and Technology, Wuhan, 430021 China; 3 Department of Neurology, Alzheimer Disease Research Center, Icahn School of Medicine at Mount Sinai, New York, NY 10029 USA; 4 Veterans Affairs Medical Center (VAPSHCS), Mental Illness Research and Educational Clinical Center, Seattle, 98108 WA USA; 5 Department of Psychiatry, University of Washington School of Medicine, Seattle, WA 98195 USA; 6 Brain and Mind Research Institute, Weill Cornell Medical College, New York, NY 10065 USA; 7 Veteran Affairs Medical Center (VAPSHCS), Geriatric Research Education and Clinical Center, Seattle, 98108 WA USA; 8 Department of Medicine and Pharmacology, University of Washington School of Medicine, Seattle, WA 98195 USA

## Abstract

The apolipoprotein E4 (ApoE4) genotype combines with traumatic brain injury (TBI) to increase the risk of developing Alzheimer’s Disease (AD). However, the underlying mechanism(s) is not well-understood. We found that after exposure to repetitive blast-induced TBI, phosphoinositol biphosphate (PIP_2_) levels in hippocampal regions of young ApoE3 mice were elevated and associated with reduction in expression of a PIP_2_ degrading enzyme, synaptojanin 1 (synj1). In contrast, hippocampal PIP_2_ levels in ApoE4 mice did not increase after blast TBI. Following blast TBI, phospho-Tau (pTau) levels were unchanged in ApoE3 mice, whereas in ApoE4 mice, levels of pTau were significantly increased. To determine the causal relationship between changes in pTau and PIP_2_/synj1 levels after TBI, we tested if down-regulation of synj1 prevented blast-induced Tau hyper-phosphorylation. Knockdown of synj1 decreased pTau levels *in vitro*, and abolished blast-induced elevation of pTau *in vivo*. Blast TBI increased glycogen synthase kinase (GSK)-3β activities in ApoE4 mice, and synj1 knockdown inhibited GSK3β phosphorylation of Tau. Together, these data suggest that ApoE proteins regulate brain phospholipid homeostasis in response to TBI and that the ApoE4 isoform is dysfunctional in this process. Down-regulation of synj1 rescues blast-induced phospholipid dysregulation and prevents development of Tau hyper-phosphorylation in ApoE4 carriers.

## Introduction

TBI is one of the most consistently identified environmental risks for late onset neurodegeneration and sporadic AD^1^. Individuals who suffer a TBI are two- to four-times more likely to develop AD^2, 3^. It was reported that ApoE4 genotype, the strongest genetic risk factor identified for sporadic AD, synergistically combines with TBI to cause a ten-fold increase in the risk of developing AD^4, 5^. Evidence suggests that the ApoE4 isoform is associated with worse outcomes after moderate to severe TBI^6^, mild TBI^7, 8^, and repetitive brain injury^9, 10^. The presence of at least one ApoE4 allele is associated with early mortality^11^, prolonged coma^12^, worse functional outcome^13–16^, and an increased risk of developing AD^5, 7^ after TBI. However, other larger studies have failed to find associations between ApoE4 and chronic traumatic encephalopathy (CTE)^17–20^. In a recent report that included a large number of community-based study subjects, TBI resulting in a loss of consciousness was not associated with AD^21^. Taken together, research on this field suggests that the pathogenic relationships between ApoE4 genotype, TBI, and the risk they collectively confer for AD and CTE have yet to be fully elucidated. Neuropathological studies of human TBI cases have described the development of neurofibrillary tangles and amyloid plaques associated with neurodegenerative processes^22, 23^. Recent studies in animal models also demonstrated TBI-linked Tau neuropathology and neurobehavioral deficits^24–27^. However, the mechanistic influence of ApoE4 genotype on developing neurodegeneration after TBI is unclear.

We have recently shown that ApoE proteins are critical determinants of brain phospholipid homeostasis and that the ApoE4 isoform is dysfunctional in this process^28^. We have demonstrated that the levels of PIP_2_ are reduced in aged human and mouse brain tissues of ApoE4 carriers as compared to levels in ApoE3 carriers. These changes are secondary to increased expression of a PIP_2_ degrading enzyme, synj1 in ApoE4 carriers. PIP_2_ is a signaling lipid involved in ion channel regulation, exocytosis, endocytosis, actin cytoskeleton rearrangement, and cell signaling^29^. PIP_2_ dephosphorylation at the synapse is mediated primarily by synj1, a phosphoinositol phosphatase that is expressed and enriched at the synapse and plays a role in endocytosis, presynaptic vesicle recycling, and postsynaptic receptor trafficking^29, 30^. Interestingly, PIP_2_ and synj1 have been recently linked to AD^31, 32^. Recently, we have shown that reduction of synj1 accelerates lysosomal clearance of Aβ in APP/PS1 transgenic mice^33^, and rescues AD-related cognitive deficits by restoring brain PIP_2_ homeostasis in ApoE4 mice^28^.

Based on these findings, we tested the hypotheses that ApoE proteins regulate changes in brain phospholipid homeostasis in response to TBI and that ApoE4-associated phospholipid dysregulation may promote development of TBI-associated amyloid and Tau pathologies. In the present study, we utilize well-established experimental models of human ApoE variants^34–37^ and a battlefield-relevant mouse model of blast-induced TBI^24–26^ to investigate the effects of ApoE isoforms on brain phospholipid composition, Aβ accumulation and Tau hyper-phosphorylation after blast-induced neurotrauma.

## Results

### Changes in hippocampal PIP_2_ levels in response to blast-induced TBI exposure in ApoE3 and ApoE4 mice

We have recently shown that ApoE4 specifically induces reduction in brain PIP_2_ levels in both aged ApoE4 human and mouse brains. However, these changes in PIP_2_ levels were not seen in young ApoE4 mouse brains by 3 months of age^28^. In this study, we investigated if blast overpressure (BOP)-induced TBI could alter brain PIP_2_ homeostasis in young ApoE4 mice when PIP_2_ changes are yet to be manifested at this age.

**Figure 1:**
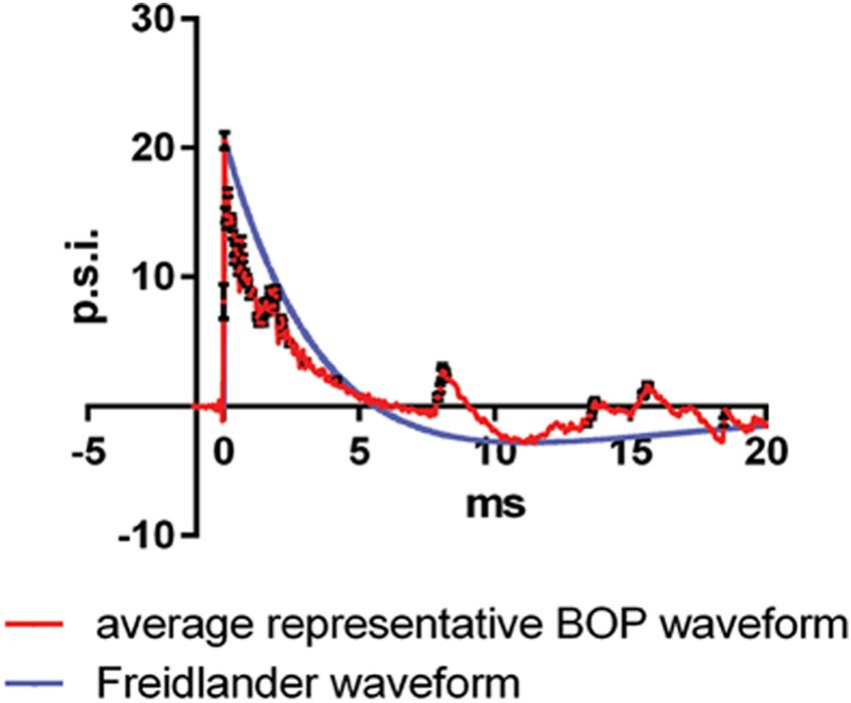
BOP waveform. The mean blast waveform from 12 representative blasts is shown in red and the expected open-field BOP waveform (Freidlander wave) shown in blue.

At 3–4 months of age, ApoE3 or ApoE4 mice were exposed to three BOP injuries over the course of three days (once per day) while briefly anesthetized each time with isoflurane (see Methods) using an established pneumatic shock tube approach^26, 38, 39^. Non-blast sham control mice received the same exposure to anesthesia as blast-exposed mice. The BOPs used for this study (Fig. 1) simulated the blast forces that would be generated by approximately 14.3 kg of trinitrotoluene (TNT) detonated in the open field at a distance of approximately 6 meters (mean peak pressure 20.0 ± 0.3 psi; primary peak duration 6.0 ± 0.1 ms; impulse 40.0 ± 10.0 psi ms). Ninety-seven percent of blast-exposed mice (37/38, 97.4%) survived the 3X treatment regimen, which was not statistically different from the survival rate of sham controls (40/40, 100%, Fisher’s Exact Test, *p* = 0.475). Immediately after recovering from anesthesia (approximately five minutes), 100% of the blast-exposed and control mice (38/38 blast-exposed and 40/40 sham controls) displayed intact visually-guided forepaw grasping responses while elevated from the tail as they were brought within reach of a foothold. In addition, all mice were monitored for two hours post-exposure (blast or sham treatments) in their home cages for adverse responses to treatment before being returned to the animal housing facility. By the end of this observation period, 97.4% of blast-exposed mice displayed normal appearing voluntary ambulation, breathing, movement responses to handling, and righting responses that by inspection were comparable to sham controls. One-hundred percent sham control and 97.4% blast-exposed mice (40/40 sham controls and 37/38 blast-exposed) survived to the conclusion of the study without veterinary intervention. Upon tissue collection, inspection of the brains revealed no indication of gross contusions or hemorrhages, including the one blast-exposed mouse that was euthanized within ten minutes after exposure to BOP due to breathing difficulty. These acute responses and experimental parameters are consistent with mild-to-moderate blast exposures in mice^39, 40^.

**Figure 2:**
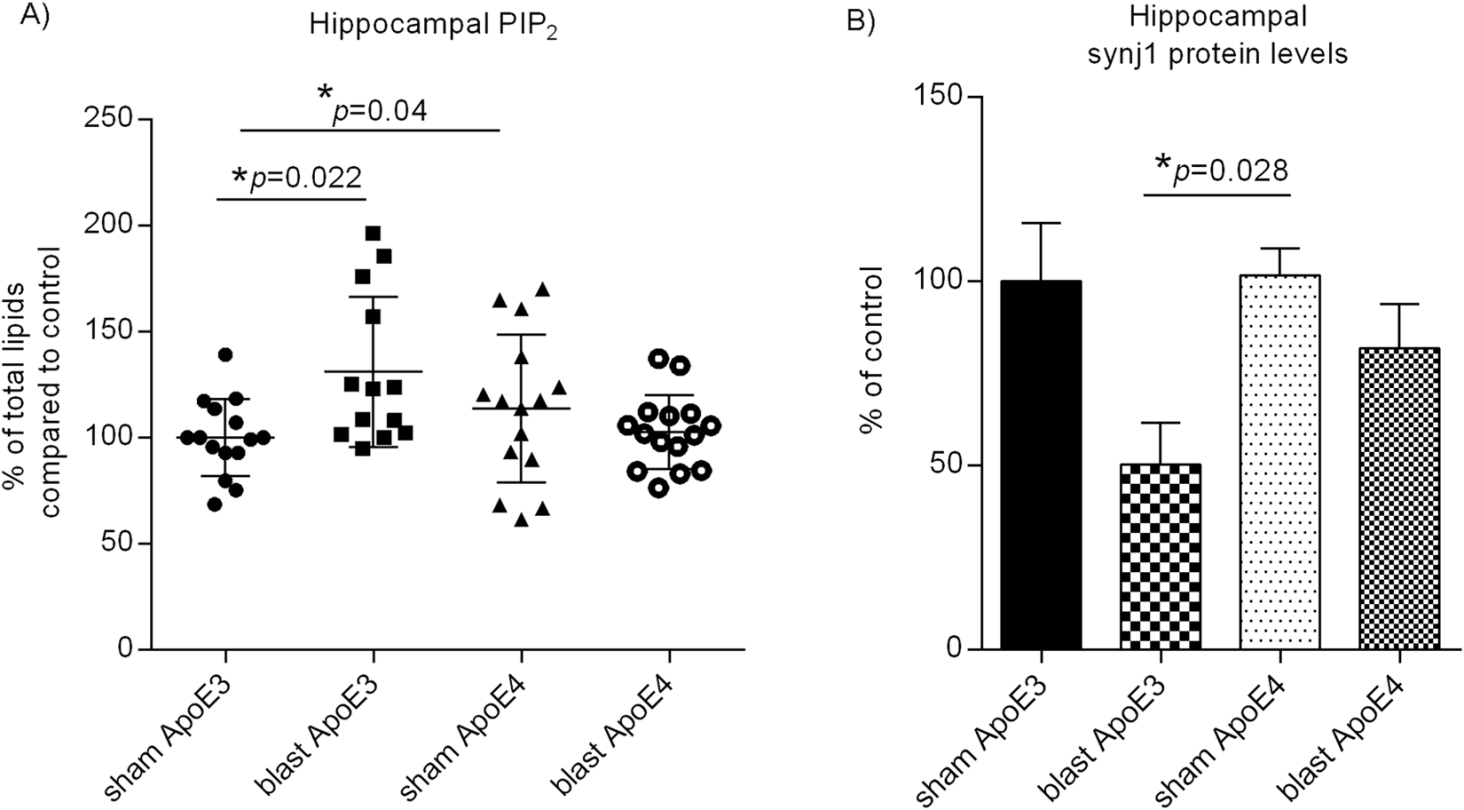
Levels of PIP_2_ and synj1 in ApoE3 and ApoE4 mouse brains in response to blast TBI. Levels of (**A**) PIP_2_, and (**B**) synj1 protein in ApoE3 and ApoE4 hippocampal regions presented as % of controls ± SEM (ApoE3 sham).

Following 3X blast exposure, the levels of hippocampal PIP_2_ in ApoE3 mice were increased 24 hours after BOP exposure (Fig. 2A: ApoE3 blast 131.0 ± 9.8% of controls, N = 13, *versus* sham 100 ± 4.7%, N = 15; one-way ANOVA F(3,54) = 3.587, *p* = 0.0194; Tukey’s multiple comparison test ApoE3 blast *versus* sham adjusted *p* = 0.022), whereas those levels in ApoE4 mouse hippocampal regions remained unchanged (ApoE4 blast 102.8 ± 4.5%, N = 15, *versus* sham 113.8 ± 9.0% of controls, N = 15; Tukey’s multiple comparison test ApoE4 blast *versus* ApoE4 sham adjusted *p* = 0.69; ApoE3 blast *versus* ApoE4 blast adjusted *p* = 0.04). No statistically significant changes were seen in levels of other phospholipids such as phosphoinositol phosphate (PIP), phosphoinositol (PI), phosphatidic acid, cardiolipin and phosphoserine in ApoE4 or ApoE3 mice 24 hours after blast TBI exposure. On the other hand, the levels of PIP_2_ were not significantly changed in neocortex, striatum or cerebellum in ApoE3 or ApoE4 mice in response to blast TBI (see Supplementary Fig. S1). No significant changes were seen in levels of other phospholipids in these brain regions. Together, these data suggest that blast exposure induces elevation of hippocampal PIP_2_ levels in ApoE3 mouse brains, whereas ApoE4 genotype blunts these changes in response to blast TBI.

### Hippocampal synj1 expression levels in ApoE3 and ApoE4 mice after blast TBI exposure

We have previously found that ApoE4 reduces brain PIP_2_ levels due to the elevated expression of the rate-limiting enzyme of brain PIP_2_ pathway, synj1^28^. Therefore, we next investigated whether blast-induced changes in brain PIP_2_ levels in ApoE3 mouse hippocampal regions are secondary to decreased expression and/or enzymatic activities of synj1. We found that no statistically significant changes were seen in levels of *synj1* mRNA in ApoE3 or ApoE4 mouse hippocampal brain regions 24 hours post blast TBI exposure (see Supplementary Fig. S2). On the other hand, there was a trend of reduction in synj1 protein levels in ApoE3 hippocampal regions 24 hours after BOP exposure (Fig. 2B: ApoE3 blast 50.3 ± 11.3% of control, N = 5, *versus* sham 100 ± 15.8%, N = 5; one-way ANOVA F(3,26) = 3.416, *p* = 0.0321; Tukey’s multiple comparison test ApoE3 blast *versus* ApoE3 sham adjusted *p* = 0.0796, ApoE3 blast *versus* ApoE4 sham adjusted *p* = 0.028). These changes are consistent with the elevation in PIP_2_ levels in ApoE3 mouse hippocampal regions after BOP exposure (Fig. 2A). There was a similar but modest trend of reduction in synj1 protein levels in ApoE4 mouse brain post blast exposure (Fig. 2B: ApoE4 blast 81.8 ± 12.0%, N = 10, *versus* sham 101.5 ± 7.3% of controls, N = 10; Tukey’s multiple comparison test ApoE4 blast *versus* sham adjusted *p* = 0.500). No significant differences were seen in levels of synj1 protein or *synj1* mRNA at baseline between ApoE3 and ApoE4 mouse brains without BOP (sham controls), which are consistent with our prior observations of ApoE mice at this age^28^. Together, these results suggest that changes in synj1 protein levels in response to blast TBI in ApoE3 mice are likely to be through translational or post-translational regulation.

### Hippocampal pTau and Tau levels in ApoE3 and ApoE4 mice after blast exposure

**Figure 3:**
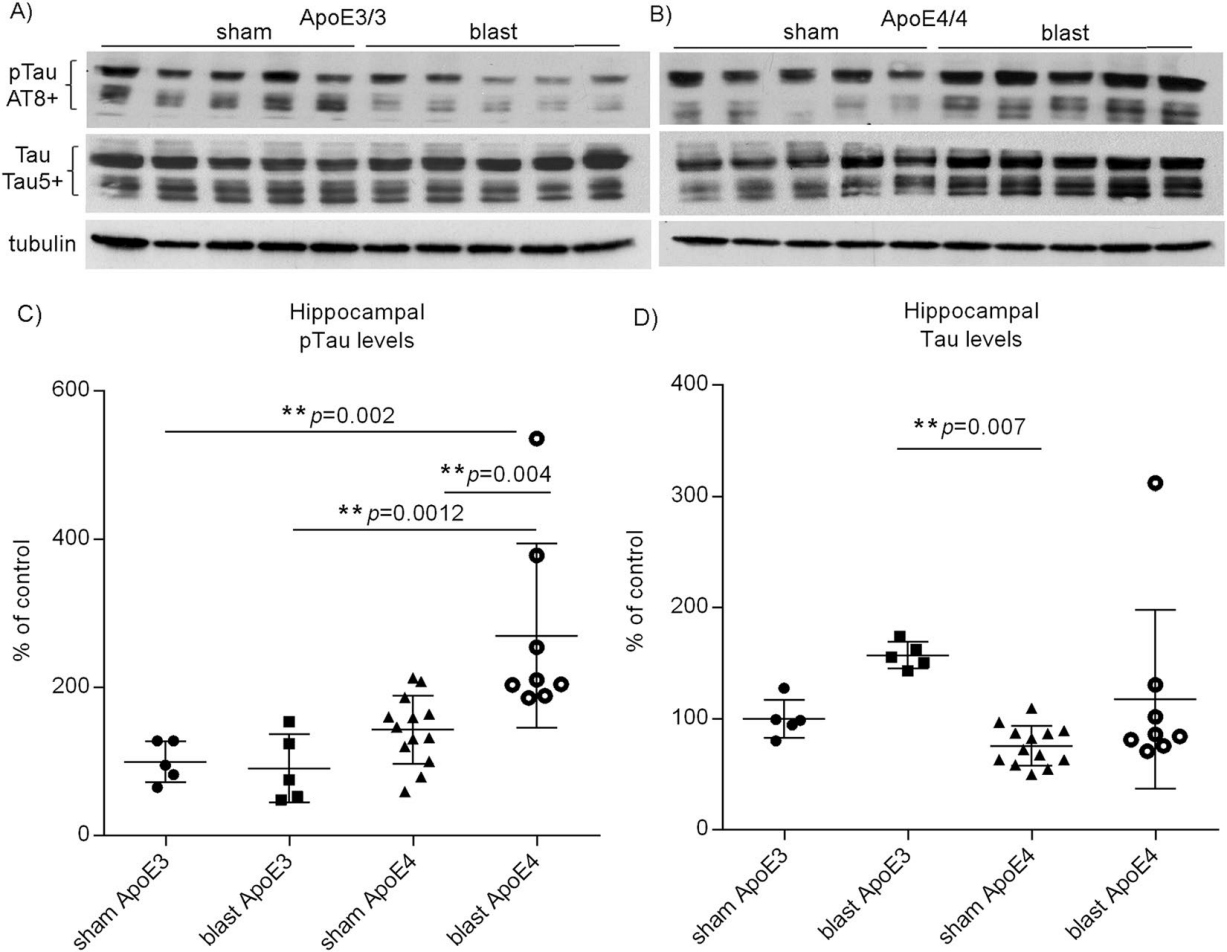
Levels of pTau and Tau in ApoE3 and ApoE4 mouse brains in response to blast TBI. A representative example of western blot of pTau and Tau in (**A**) ApoE3 and (**B**) ApoE4 mouse brains. Levels of (**C**) pTau and (**D**) Tau are presented as % of controls ± SEM (ApoE3 sham).

Following blast exposure, the levels of pTau determined by AT8 antibodies using western blot analysis were significantly elevated 24 hours post-BOP in ApoE4 hippocampal brain regions (Fig. 3B and C: ApoE4 blast 270.4 ± 44.0%, N = 8 *versus* ApoE4 sham 143.3 ± 12.8% of controls, N = 13; one-way ANOVA F(3,27) = 8.800, *p* = 0.0003; Tukey’s multiple comparison test ApoE4 blast *versus* ApoE4 sham adjusted *p* = 0.004), whereas no significant changes in levels of pTau were seen in ApoE3 mouse brains (Fig. 3A and C: ApoE3 blast 91.2 ± 20.7%, N = 5 *versus* ApoE3 sham 100 ± 12.5% of controls, N = 5; Tukey’s multiple comparison test: ApoE3 blast *versus* ApoE3 sham adjusted *p* = 0.99, ApoE3 blast *versus* ApoE4 blast adjusted *p* = 0.0012, ApoE3 sham *versus* ApoE4 blast adjusted *p* = 0.002). Similar changes were observed using an antibody against pTau at Ser202 residue (CP13; data not shown). On the other hand, levels of total Tau determined by Tau5 antibodies were increased in ApoE3 hippocampal brain regions after BOP exposure (Fig. 3A and D: ApoE3 blast 157.2 ± 5.3%, N = 5 *versus* ApoE3 sham 100 ± 7.7% of controls, N = 5; one-way ANOVA F(3,27) = 4.626, *p* = 0.0098; Tukey’s multiple comparison test: ApoE3 blast *versus* ApoE3 sham adjusted *p* = 0.19, ApoE3 blast *versus* ApoE4 sham adjusted *p* = 0.007), whereas those levels in ApoE4 mouse brains were only modestly increased without statistical significance (Fig. 3B and D: ApoE4 blast 117.7 ± 28.5%, N = 8 *versus* ApoE4 sham 75.6 ± 5.0%, N = 13; Tukey’s multiple comparison test: ApoE4 blast *versus* ApoE4 sham adjusted *p* = 0.16). Interestingly, at baseline without BOP exposure, there was a trend of elevation in pTau levels in ApoE4 hippocampal brain regions as compared to ApoE3 counterparts (Fig. 3C: ApoE4 sham 143.3 ± 12.8%, N = 13 *versus* ApoE3 sham 100 ± 7.7%, N = 5; F(3,27) = 8.800; Tukey’s multiple comparison test adjusted *p* = 0.68), whereas levels of total Tau in ApoE4 brains were lower than those in ApoE3 mice (Fig. 3D: ApoE4 sham 75.6 ± 5.0%, N = 13 *versus* ApoE3 sham 100 ± 7.7%, N = 5, F(3,27) = 4.626; Tukey’s multiple comparison test adjusted *p* = 0.71). These results suggest that ApoE isoforms have differential effects on pTau/Tau processing, and that ApoE4 genotype may exacerbate Tau hyper-phosphorylation in the presence of blast exposure. These patterns of changes in pTau and total Tau levels in response to BOP-induced TBI were only seen in hippocampal brain regions, but not in cortical, striatal or cerebellar brain regions, suggesting regional vulnerability of hippocampi in response to blast TBI.

We also investigated the effects of ApoE4 and TBI on Aβ accumulation and ApoE secretion, and found that there were no significant changes in levels of mouse Aβ_40_ or Aβ_42_, or in levels of secreted human ApoE in ApoE4 and ApoE3 hippocampal regions after BOP exposure (see Supplementary Fig. S3).

### Reduction of synj1 decreases pTau levels in N2a cells

**Figure 4:**
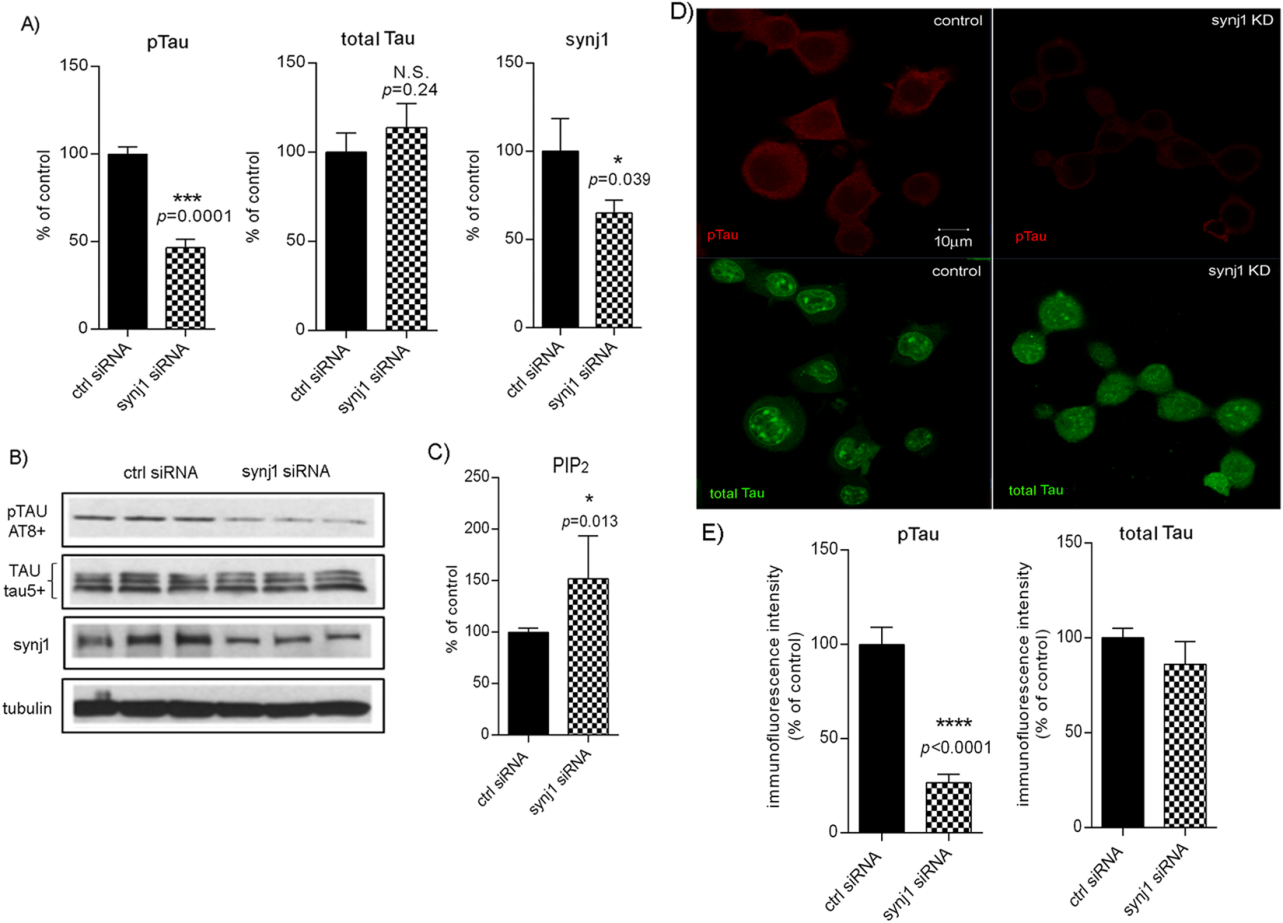
Reduction of pTau with synj1 knockdown *in vitro*. (**A**) The levels of pTau, Tau and synj1 protein in N2a cells after synj1 siRNA. Results are presented as % of levels in controls ± SEM. (**B**) A representative example of western blot analysis. (**C**) PIP_2_ levels. (**D**) Immunofluorescence staining of pTau (red) and Tau (green) with (**E**) quantification.

We have demonstrated that there were differential changes in hippocampal PIP_2_/synj1 levels, as well as pTau/Tau levels in young ApoE3 versus ApoE4 mice after BOP exposure. Therefore, we determined whether there is a causal relationship between blast TBI-induced changes in PIP_2_/synj1 levels and changes in pTau/Tau levels. We first investigated the effects of down-regulation of synj1 on Tau phosphorylation *in vitro*. Using a N2a cell line stably transfected with human wild-type Tau, we found that levels of pTau were significant reduced (Fig. 4A and B: 53.5% of reduction, T(4) = 14.79, *p* = 0.0001) with synj1 knockdown (KD) by synj1 siRNA, whereas levels of total Tau were slightly elevated although changes did not achieve statistical significance (113.8% of controls, T(4) = 1.365, *p* = 0.2441). The levels of holoAPP were not changed with synj1 siRNA treatment, which is consistent with our previous observations^33^. Down-regulation of synj1 in N2a cells led to a significant elevation in PIP_2_ levels (Fig. 4C: 151.8% of controls, T(10) = 3.015, *p* = 0.013), similarly to our prior findings^28, 33^.

Moreover, confocal microscopy examination of N2a cells suggests that the amount of pTau (red fluorescent signals) recognized by AT8 antibodies was significantly reduced with synj1 knockdown (Fig. 4D top panels and 4E; 26.8% of controls, T(11.64) = 7.292, *p* < 0.0001), whereas the amount of total Tau (green fluorescent signals) recognized by Tau5 antibodies was not dramatically changed (Fig. 4D bottom panels and 4E; 86.0% of controls, T(10.78) = 1.056, *p* = 0.314). It was also noted that with reduction of pTau (mainly in cytosol) in synj1 KD conditions, the amount of cytosolic total Tau was increased as compared to controls. The reduction of synj1 expression in KD conditions was confirmed by fluorescent signals recognized by synj1 antibodies (see Supplementary Fig. S4).

### Down-regulation of synj1 prevents elevation of pTau levels in ApoE4 mice in response to blast TBI exposure

**Figure 5:**
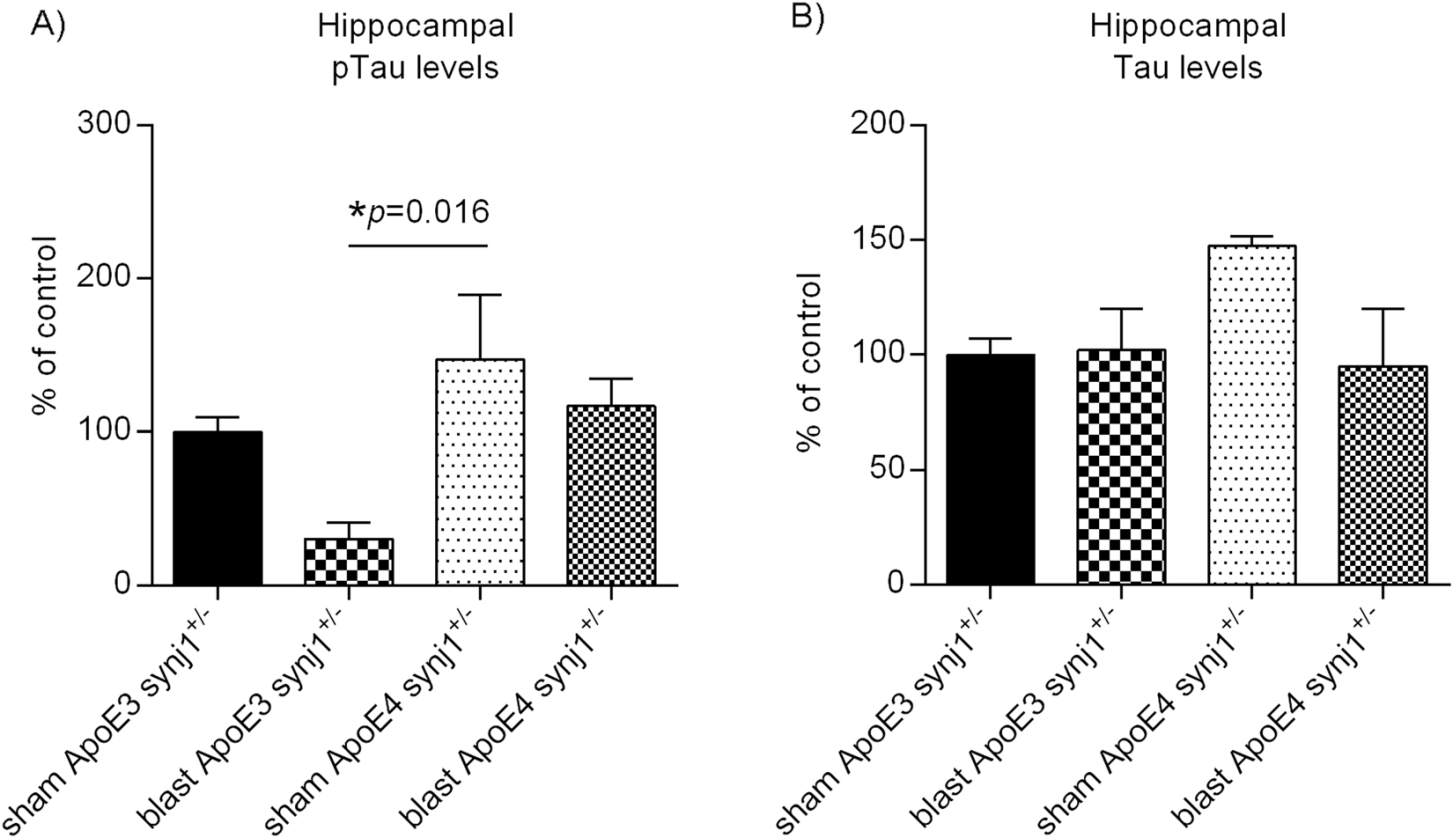
Down-regulation of synj1 prevents pTau elevation in ApoE4 mice after blast TBI. The levels of (**A**) pTau and (**B**) total Tau are presented as % of controls ± SEM (ApoE3 synj1^+/−^ sham).

We next determined if down-regulation of synj1 *in vivo* could ameliorate blast TBI-induced tau hyper-phosphorylation in ApoE4 mice. As shown in Fig. 5A, pTau levels after blast TBI exposure were reduced in ApoE3 synj1^+/−^ mice but did not achieve statistical significance (N = 5/group; ApoE3 synj1^+/−^ blast 30.6 ± 10.4% of controls *versus* sham 100 ± 9.7%; one-way ANOVA F(3,16) = 4.220, *p* = 0.0223; Tukey’s multiple comparison test ApoE3 blast *versus* ApoE3 sham adjusted *p* = 0.21, ApoE3 blast *versus* ApoE4 sham *p* = 0.016), whereas elevation of pTau in ApoE4 mice after blast exposure was completely abolished with a trend of reduction in pTau levels in ApoE4 mice with synj1 knockdown (ApoE4 synj1^+/−^ blast 116.6 ± 17.9% *versus* sham 147.3 ± 42.3% of controls; F(3,16) = 4.220; Tukey’s multiple comparison test: ApoE4 blast *versus* ApoE4 sham adjusted *p* = 0.80, ApoE3 sham *versus* ApoE4 sham *p* = 0.52). On the other hand, levels of total Tau were not changed in ApoE3 synj1^+/−^ mice after blast exposure (Fig. 5B: ApoE3 synj1^+/−^ blast 102.1 ± 17.8% of controls; one-way ANOVA F(3,16) = 2.357, *p* = 0.11; Tukey’s multiple comparison test adjusted *p* = 0.9997), whereas levels of total Tau in ApoE4 synj1^+/−^ mice in sham group were much higher than other experimental groups such as ApoE3 sham (ApoE4 synj1^+/−^ sham 147.3 ± 4.3% of controls *versus* ApoE3 synj1^+/−^ sham 100 ± 9.7%; adjusted *p* = 0.19) with a trend of reduction after blast exposure (ApoE4 synj1^+/−^ blast 95.0 ± 24.9% of control *versus* ApoE4 synj1^+/−^ sham 147.3 ± 4.3% of controls; adjusted *p* = 0.13). There were no significant changes in brain synj1 protein or PIP_2_ levels in response to blast TBI exposure in both ApoE3 synj1^+/−^ and ApoE4 synj1^+/−^ mice (see Supplementary Fig. S5).

Together, our results suggest that synj1 down-regulation abolishes dysregulation of PIP_2_ levels and hyper-phosphorylation of Tau in response to blast injury exposure in ApoE4 mice.

### Blast induces GSK3β activation in ApoE4 mice and synj1 knockdown reduces pTau partially through inactivation of GSK3β

**Figure 6:**
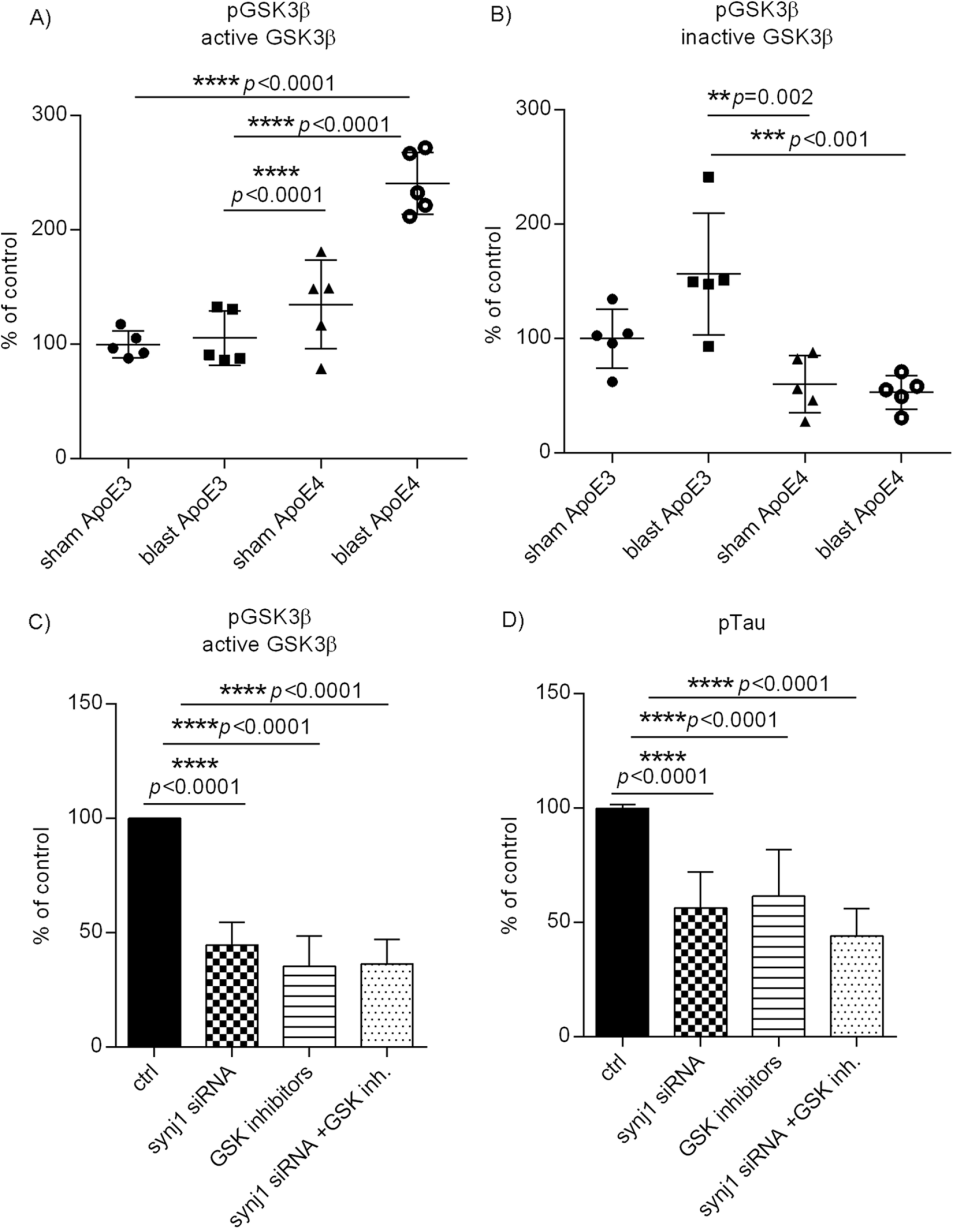
Blast induces GSK3β activation in ApoE4 mice and synj1 knockdown reduces pTau through inactivation of GSK3β. Levels of (**A**) active and (**B**) inactive pGSK3β after blast. Results are presented as % of controls ± SEM (ApoE3 sham). Levels of (**C**) active pGSK3β and (**D**) pTau in N2a cells with synj1 siRNA, GSK inhibitors, or combination. Results are presented as % of controls ± SEM.

We next determined whether blast injury induces any changes in the levels of two common Tau phosphorylation kinases: GSK3β and cyclin dependent kinase 5 (CDK5) in ApoE3 and ApoE4 mouse brains. As shown in Fig. 6A after blast exposure, levels of active GSK3β (pGSK3β recognized by an antibody against Y216)^41^ were significantly increased in ApoE4 mice (ApoE4 sham 134.9 ± 38.6%, ApoE4 blast 241.0 ± 27.0%; N = 5/group, one-way ANOVA F(3,16) = 29.36, *p* < 0.0001), whereas levels of inactive GSK3β (pGSK3β recognized by antibody at Ser9)^42^ were higher in ApoE3 mice but did not achieve statistical significance (Fig. 6B: ApoE3 sham 100 ± 25.8%, ApoE3 blast 156.5 ± 53.4%; N = 5/group, one-way ANOVA F(3,16) = 10.34, *p* = 0.0005, Turkey’s comparison test: ApoE3 sham *versus* ApoE3 blast adjust *p* = 0.067). There were trends of higher basal levels of active GSK3β and lower basal levels of inactive GSK3β in ApoE4 sham mice compared to ApoE3 counterparts, but these differences did not achieve statistical significance (active GSK3β: ApoE4 sham 134.9 ± 38.6% *versus* ApoE3 sham 100.0 ± 11.8%, adjusted *p* = 0.22; inactive GSK3β: ApoE4 sham 60.11 ± 25.1% *versus* ApoE3 sham 100.0 ± 25.8%, adjusted *p* = 0.26). However, there were no significant changes in total GSK3β levels of ApoE4 or ApoE3 mouse brains in response to blast TBI exposure (ss Supplementary Fig. S6A and S6B: Tukey’s multiple comparison test: ApoE4 blast *versus* ApoE4 sham adjusted *p* = 0.90, ApoE3 sham *versus* ApoE4 sham *p* = 0.21). On the other hand, the levels of CDK5 were not significantly changed in ApoE4 mice in response to blast exposure, whereas there was a trend of increased CDK5 levels in ApoE3 mice after BOP exposure (see Supplementary Fig. S6C). It was noted that basal levels of CDK5 in ApoE4 sham mouse brains were higher than those of ApoE3 counterparts.

We next investigated if synj1 down-regulation reduces Tau phosphorylation through inhibition of either of these two Tau kinases (GSK3β and CDK5). As shown in Fig. 6C, synj1 knockdown inhibited GSK3β activities with reduced levels of pGSK3β at Y216 (44.7 ± 9.9% of controls; N = 4–9/group, one-way ANOVA F(3,23) = 47.96, *p* < 0.0001), similarly to treatment with GSK3β inhibitors (indirubin or kenpaullone; 35.4 ± 13.3% of controls; adjusted *p* < 0.0001). However, the combination of synj1 knockdown and GSK3β inhibitors failed to further reduce GSK3β activities (36.4 ± 10.7% of controls; Tukey’s multiple comparison test: combined *versus* control *p* < 0.0001; combined *versus* synj1 alone *p* = 0.57; combined *versus* GSK inhibitors alone *p* = 0.997).

Consistently, treatment with synj1 knockdown (62.2 ± 18.4% of controls; N = 10–18/group, one-way ANOVA F(3,54) = 11.84, *p* = 0.0012; Tukey’s multiple comparison test adjusted *p* < 0.0001), or GSK3 inhibitors (66.0 ± 22.9% of controls; adjusted *p* = 0.0006) reduced pTau levels, but the combination of synj1 knockdown and GSK3β inhibitors failed to further reduce pTau levels (Fig. 6D: 52.0 ± 35.2% of controls; Tukey’s multiple comparison test: combined *versus* control *p* < 0.0001; combined *versus* synj1 alone *p* = 0.70; combined *versus* GSK inhibitors alone *p* = 0.32), suggesting that the effects of synj1 reduction on Tau phosphorylation are mediated, at least partially, through inactivation of GSK3β. No significant changes were seen in levels of total GSK3β, total Tau or CDK5 in any of these treatments (see Supplementary Fig. S7).

## Discussion

The ApoE-genotype dependent responses to TBI have been studied with many mechanisms proposed including alterations in neurogenesis, cholesterol trafficking, amyloid deposition and cholinergic dysfunction^43, 44^. Our study investigates the association between a known genetic susceptibility factor (ApoE4) and an environmental risk factor (TBI) for the development of neurodegeneration and AD. We demonstrate that there are ApoE-genotype specific changes in brain PIP_2_ homeostasis in response to blast injury, and that such changes contribute to the development of Tau pathology after blast. Therefore, our findings (as summarized in Table 1) are the first mechanistic studies that link ApoE4 genotype-specific changes in brain phospholipid homeostasis to the development of neurodegenerative processes after blast-induced TBI in ApoE4 carriers. These results implicate a novel mechanism by which ApoE4 genotype promotes the development of neurodegeneration after TBI.

**Table 1:** Summary of changes in different ApoE mice in response to blast TBI.

**wild-type synj1 levels**			
		**PIP**_**2**_	**synj1**
ApoE3	blast vs sham		
ApoE4	blast vs sham	≈	≈
**wild-type synj1 levels**			
		**phospho-Tau**	**total Tau**
ApoE3	blast vs sham	≈	
ApoE4	blast vs sham		≈
**partial synj1 reduction**			
		**phospho-Tau**	**total Tau**
ApoE3 (synj1^+/−^)	blast vs sham		≈
ApoE4 (synj1^+/−^)	blast vs sham	≈	≈

Blast induces PIP_2_ elevation and synj1 reduction in ApoE3 but not in ApoE4 mice. Blast induces Tau elevation with no changes in p-Tau in ApoE3 mice, whereas blast induces p-Tau elevation with no changes in Tau in ApoE4 mice. In ApoE3 synj1^+/−^ mice, pTau is reduced after blast, whereas blast-induced pTau elevation is abolished in ApoE4 synj1^+/−^ mice.

Our study examines the effect of repetitive blast-TBI in the context of well-established genetic risk factors for Alzheimer’s disease. In designing the current study we had concerns that a single blast exposure would more likely yield minimally interpretable negative results, as well as being less relevant to the exposure conditions of the Veterans we seek to model in mice. Single mild TBI exposures are typically reported to develop transient and relatively low levels of Alzheimer’s-associated pathology. Conversely, repetitive TBI has gathered significant attention for its strong association with more persistent pathology, such as synapse loss, vasculopathy and Tau burden, which contributes to development of Alzheimer’s disease. Studies by others^43^ support the notion that significant and long-lasting ApoE-associated phospholipid changes are greater with more severe TBI injuries.

In addition, we have considerable experience at this point in the effects of single versus multiple blast exposures. Overall, we have found that one blast exposure causes modest and short-lived effects on neuropathology and behavior. For example, we found that at 30 days post-exposure a single blast has no residual effect on performance on the rotarod, whereas 3 blast exposure does^38^. More importantly, this is a positive translational implication using this experimental paradigm, in that persistent behavioral and neurological effects are most consistent with findings among Veterans who have experienced repetitive blast-related mTBIs. Therefore, we examined how repetitive mild TBI produces similar Alzheimer’s disease-associated changes in manners dependent on ApoE genotypes and synj1 expression.

Our data support specific effects of both TBI and ApoE isoforms in hippocampus, which are consistent with previous studies^43, 45^. We have shown that changes in PIP_2_ homeostasis and pTau levels are most dramatic in hippocampal regions of ApoE3 and ApoE4 mice, suggesting specific vulnerability of hippocampal regions in response to TBI. Several hypotheses have been proposed in relation to hippocampal vulnerability following TBI such as direct biomechanical forces, excitotoxic neuropathological changes, vascular compromise, metabolic changes, and/or transneuronal degeneration^46, 47^. Evidence also suggests that injuries resulting from blast are distinct from those arising from blunt force or other types of TBI^48^. Blast exposure produces intense shock waves that may injure brain tissue by various biophysical means that distort vascular and/or neural tissue, and produce inertial injury from rapid acceleration/deceleration of the brain^49, 50^. It is possible that hippocampal brain regions are more susceptible to blast force induced injury due to heightened anatomical and vascular susceptibility^51^. On the other hand, it is possible that hippocampal PIP_2_ is among the most sensitive lipid species in the brain, where levels are altered in response to a wide array of central nervous system insults including trauma, ApoE4 genotype^28^, and other factors known to promote neurodegenerative processes. Our results suggest that elevation of brain PIP_2_ through reduction of synj1 expression could be a potential therapeutic strategy to prevent the development of neurodegeneration after TBI, and to possibly improve clinical outcomes of those affected by TBI-AD particularly in ApoE4 carriers.

Interestingly, our results suggest differential effects of ApoE genotype on development of Tau hyper-phosphorylation after blast TBI, which could be modulated by brain synj1 expression and PIP_2_ levels. We are currently investigating whether down-regulation of synj1 with resultant elevation of brain PIP_2_ levels in ApoE4 mice is sufficient to protect brains from development of long-term neurodegenerative processes. The regulation of Tau phosphorylation by PIP_2_/synj1 is at least partially mediated through inhibition of GSK3β (Fig. 6). The crosstalk between PIP_2_ and GSK3β has been described previously in insulin signaling pathways by which insulin activates PI-3-kinase that converts PIP_2_ to phosphoinositol triphosphate (PIP_3_) which subsequently activates protein kinase B (PKB) leading to phosphorylation and inhibition of GSK3β^52^. It is possible that reducing synj1 prevents dephosphorylation of PIP_2_ which could favor the generation of PIP_3_ from PIP_2_ and activate downstream signaling by PKB to inhibit GSK3β and reduce Tau phosphorylation.

It should be noted that we did not see any differences in the levels of Aβ_40_ and Aβ_42_ between blast *versus* sham conditions in our ApoE4 or ApoE3 mice (see Supplementary Fig. S3A and S3B). It is well established that ApoE plays a critical role in mediating Aβ clearance^53, 54^. Overall, 10% of non-ApoE4, 35% of heterozygous ApoE4, and 100% of homozygous ApoE4 cases present with Aβ plaques after TBI^55^. We speculate that the differences in Aβ accumulation and clearance in different ApoE genotypes after TBI may take longer to develop. Alternatively, it is possible that we examined endogenous mouse Aβ levels, which are relatively low. To study amyloid plaque pathologies induced by TBI, we may need to utilize ApoE mice with an AD transgenic background. Moreover, we did not observe any changes in ApoE secretion in ApoE3 or ApoE4 mouse brains after blast (see Supplementary Fig. S3C), which does not support the hypothesis that differential effects of ApoE genotypes on brain lipids may be mediated through differences in secreted ApoE levels after TBI. It should also be noted that we used all male ApoE mice in our study to avoid any potential gender confounding effects on our results. However, previously published epidemiologic and neuroimaging data suggest that ApoE4 has a stronger effect in promoting AD risk in females than in males^56^. It would be interesting in the future studies to perform comparative evaluations in both male and female mice after TBI exposure and determine any sex-specific impact on blast-induced phospholipid dysregulation development of Tau hyper-phosphorylation in different ApoE groups.

One interesting aspect based on our results is that the influence of ApoE4 on responses to TBI may be a loss of reparative function rather than a gain of negative function. We speculate that elevated PIP_2_ levels in ApoE3 mice after blast exposure may function as a protective mechanism to activate repair machinery, and ameliorate injury-related neurodegenerative processes like Tau hyper-phosphorylation. In contrast, the ApoE4 genotype blunts any changes in brain PIP_2_ and synj1 in response to blast TBI which may contribute to the development of ApoE4-induced Tau hyper-phosphorylation.

It should be noted that reduction in synj1 protein levels in ApoE3 mouse hippocampal regions in response to blast TBI exposure is not likely due to transcriptional regulation because changes in *synj1* mRNA levels were not seen (see Supplementary Fig. S2). Since synj1 protein is highly enriched at the synapse^29, 30^, we speculate that these changes in synj1 protein levels post-BOP occur at local synaptic terminals, possibly through post-translational regulation such as accelerated degradation of synj1 proteins in response to blast TBI. We have previously shown that the ApoE4 genotype perturbs neuronal, especially synaptic PIP_2_ homeostasis, by altering synj1 expression at synapses, which could contribute to development of ApoE4-induced cognitive deficits and synaptic dysfunction^28^. Interestingly, key neuropathological features of AD development as late complications of TBI include hippocampal neuron loss and synaptic dysfunction^57–59^. Therefore, it is possible that TBI may exacerbate ApoE4-associated synaptic PIP_2_ dyshomeostasis, which could contribute to TBI-induced neurodegenerative processes.

In recent years, phospholipid profiles in plasma and cerebrospinal fluid samples of TBI patients^60, 61^, and in ApoE mouse brains^62, 63^ have been studied. To our knowledge, no report to date has examined phospholipid profiles of ApoE mouse models after blast TBI. Our studies had a specific focus on low abundance phosphoinositol species such as PIP and PIP_2_^28, 32, 33^, and indicated that hippocampal PIP_2_ levels were lower (Fig. 2A) after blast TBI in ApoE4 mouse brain regions, associated with a trend to higher PI levels, as compared to sham controls or to their ApoE3 counterparts after blast exposure. It is possible that more drastic changes in PI/PIP/PIP_2_ homeostasis of ApoE4 mouse brains may occur during chronic phases of blast injury. Studies are currently ongoing to investigate the effects of ApoE4 and TBI on brain phospholipid profiles at subacute and chronic stages of TBI.

In summary, our results in this study suggest that ApoE4 genotype specific PIP_2_ dyshomeostasis contributes to the development of Tau hyper-phosphorylation after blast TBI exposure, and that elevation of brain PIP_2_ levels by reducing synj1 expression may ameliorate TBI- associated Tauopathy in ApoE4 carriers.

## Methods

### Mouse models

The human ApoE4 or ApoE3 knock-in mouse models (RRID: IMSR_TAC:2542 and 1549) in which the coding region of human *ApoE* gene replaces that of the mouse *ApoE* gene^34–36^, were purchased from Taconic Biosciences Inc. under license agreements for generation of synj1 haploinsufficiency mice with either a human ApoE4 or ApoE3 background. To generate these mice, the ApoE4/4 or ApoE3/3 KI mice were mated with heterozygous synj1 null mice (synj1^+/−^) to generate offspring that express human ApoE4/4 or E3/3 with synj1^+/−^ or synj1^+/+^ as described previously^28^. Four genotypes were used in this study: ApoE3/3 synj1^+/+^, ApoE3/3 synj1^+/−^, ApoE4/4 synj1^+/+^, ApoE4/4 synj1^+/−^. Males were used in this study to avoid potential confounding effects of interactions between genders and ApoE genotypes.

### Blast overpressure (BOP) exposure

Experimental blast exposure was performed using a pneumatic shock tube designed to recapitulate open-field blast forces caused by explosive detonation as described previously^26, 38^. Briefly, all mice (blast-exposed and sham controls) were anesthetized using an isoflurane gas anesthesia system and placed onto a restraint grid in the shock tube in a supine orientation for approximately 3–5 minutes. Blast-exposed mice received BOP exposures (mean peak pressure: 20.0 ± 0.30 pounds per square inch (psi), primary peak duration: 6.0 ± 0.1 milliseconds (ms), impulse: 40.0 ± 10.0 psi · ms); once per day for three consecutive days followed by brain tissue harvest 24 hours after the last blast. Of the 38 blast-exposed mice used in this study only 1 was euthanized for humane reasons prior to study endpoint. All animal procedures were approved by The Institutional Animal Care and Use Committee (IACUC) of JJP VAMC and VAPSHCS and performed in accordance with the guidelines of Association for Assessment and Accreditation of Laboratory Animal Care (AAALAC).

### Brain lysate preparation and analysis

Snap-frozen mouse brains (hippocampal, neocortical, striatal and cerebellar regions) were homogenized in lysis buffer and processed via step-wise solubilization^64, 65^, followed by sodium dodecyl sulphate-polyacrylamide gel electrophoresis (SDS-PAGE) to determine levels of pTau, total Tau, synj1, GSK3β, pGSK3β, β-actin and tubulin. Levels of Aβ_40_ and Aβ_42_ were determined by high-sensitive mouse Aβ_40_ and Aβ_42_ enzyme-linked immunosorbent assay (ELISA) kits (Wako). Levels of ApoE in brain lysates were determined by human ApoE ELISA kit (ABCAM, Inc.).

### Phospholipid analysis

Blast or sham exposed mouse brain samples were used for lipid extraction, followed by anion-exchange high pressure liquid chromatography quantification as described previously^33, 66^.

### Message RNA (mRNA) extraction

mRNA was extracted using an RNeasy spin column kit (Qiagen). Levels of *synj1* mRNA were determined by quantitative Real time polymerase chain reaction. Controls included mouse 18S ribosomal subunit and glyceraldehyde-3-phosphate dehydrogenase (GADPH).

### Cell lines and immunocytochemical studies

Mouse N2a neuroblastoma cells stably transfected with cDNA encoding human wildtype Tau441 were maintained in medium containing 50% DMEM and 50% OPTI-MEM, supplemented with 5% fetal bovine serum, antibiotics, and 200 µg/ml G418 (Invitrogen). N2a cells were transfected with synj1 small interfering RNA (siRNA) and maintained for 4–5 days to achieve ~50–80% knockdown of synj1 protein levels as previously described^33^ at which time SDS-PAGE and western blotting were performed to determine levels of pTau, total Tau, synj1, holo amyloid precursor protein (APP), β-actin and tubulin. In some experiments, cells transfected with synj1 siRNA were treated with a GSK3 inhibitor (indirubin at 5 µM or kenpaullone at 10 µM; Sigma), or vehicle control (DMSO) for 24 hours before subjected for analysis of pTau, Tau, pGSK3β and GSK3β levels. Alternatively, cells transfected with siRNA were fixed and stained for imaging analysis using a Zeiss LSM510 confocal microscope. Levels of pTau and total Tau were determined by immunostained with AT8 antibodies followed by Texas red anti-mouse immmunoglobulin (IgG), and then sequentially immunostained with Tau5 antibodies followed by Alexa^488^ conjugated anti-mouse IgG. In parallel, levels of synj1 were determined by immunostained with synj1 antibody followed by Texas red anti-rabbit immunoglobulin (IgG). The immunofluorescence intensity was quantified after normalized by DAPI signals.

### Antibodies and reagents

The following commercially available antibodies were used: anti-pTau (AT8; pSer202, pThr205) and total Tau (Tau 5) from ThermoFisher (RRID:AB_223647 and 10980631), anti-synj1 (rabbit polyclonal Ab, Novus; RRID:AB_11047653), anti-β actin and tubulin (Santa Cruz; RRID:AB_476697 and 477498), anti-holoAPP (MAB348, Millipore; RRID:AB_94882), anti-GSK3β (rabbit monoclonal Ab, Cell Signaling; RRID:AB_490890), anti-pGSK3β (Ser9, inactive form of GSK3β, rabbit polyclonal Ab, Cell Signaling; RRID:AB_331405), anti-pGSK3β (Y216, active form of GSK3β, rabbit polyclonal Ab, Abcam; RRID:AB_1310290), anti-mouse and rabbit horse radish peroxidase, Texas-Red or Alexa^488^ conjugated anti-mouse IgG (ThermoFisher; RRID:AB_2556542, 2540618, 10374713, 10983944, 2535987 and 1090271), were purchased. The pAb369 antibody which recognizes the C-terminus of APP was used to detect mouse holo-APP and c-terminal fragments^67^.

### Statistical analysis

Levels of synj1, pTau and total Tau, pGSK3β (inactive and active forms) and total GSK3β were normalized to tubulin levels and expressed as a percentage of control (±SEM). Absolute Aβ_40_, Aβ_42_ and ApoE concentrations were quantitatively determined by ELISA (Wako and ABCAM, Inc.) and expressed as a percentage of control (±SEM). Levels of pTau, total Tau and synj1 immunofluorescence signals were normalized to DAPI levels and expressed as a percentage of control (±SEM). Independent-sample *t* tests were used to determine significant mean differences (the threshold for significance sets at *p* < 0.05, two-tailed). To determine the overall statistical significance of differences between multiple groups, analysis of variance (ANOVA) was performed followed by post-hoc Tukey tests to determine the significance of differences between pairs of means. All statistical analyses were performed using GraphPad Prism 6.0 (GraphPad Software, Inc.).

## Electronic supplementary material

Supplementary Figures
