# Supplementary material for: ApoE4-associated phospholipid dysregulation contributes to development of Tau hyper-phosphorylation after traumatic brain injury
Source: Sci Rep. 2017 Sep 12;7:11372. doi: 10.1038/s41598-017-11654-7 (PMC5595858; doi:10.1038/s41598-017-11654-7)
Supplement: Supplementary file 1 — Supplementary Figures [file 41598_2017_11654_MOESM1_ESM.pdf]

ApoE4-associated phospholipid dysregulation contributes to development of Tau hyper-phosphorylation after traumatic brain injury Jiqing Cao, Farida El Gaamouch, James S. Meabon, Kole D. Meeker, Li Zhu, Margaret B. Zhong, John Bendik, Gregory Elder, Ping Jing, Jiahong Xia, Wenjie Luo, David G. Cook, Dongming Cai

Supplementary Figures

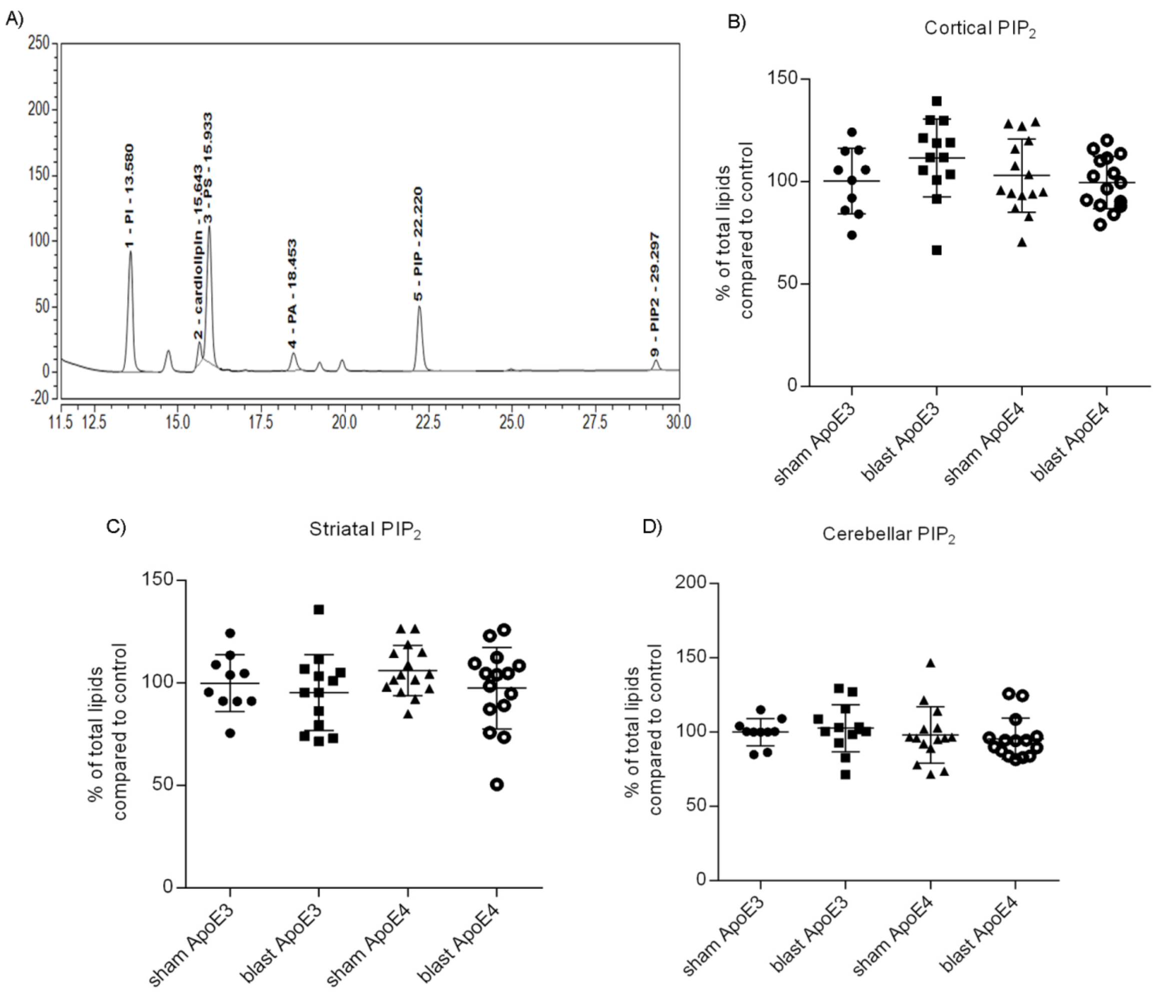

Figure 1 The PIP_2 levels in various brain regions of ApoE3 and ApoE4 mice after blast TBI. A) A typical example of HPLC chromatogram showing phospholipid peaks including phosphoinositol (PI), cardiolipin, phosphoserine (PS), phosphatidic acid (PA), phosphoinositol phosphate (PIP) and phosphoinositol biphosphate (PIP_2). Levels of PIP_2 in B) neocortical, C) striatal and D) cerebellar brain regions are presented as % of controls ±SEM (ApoE3 sham). ApoE3 sham N=10, ApoE3 blast N=13, ApoE4 sham N=15, ApoE4 blast N=15. B) one-way ANOVA $F(3,49)=1.443$, $p=0.242$, C) one-way ANOVA $F(3,49)=1.120$, $p=0.350$, and D) one-way ANOVA $F(3,49)=0.528$, $p=0.665$.

ApoE4-associated phospholipid dysregulation contributes to development of Tau hyper-phosphorylation after traumatic brain injury Jiqing Cao, Farida El Gaamouch, James S. Meabon, Kole D. Meeker, Li Zhu, Margaret B. Zhong, John Bendik, Gregory Elder, Ping Jing, Jiahong Xia, Wenjie Luo, David G. Cook, Dongming Cai

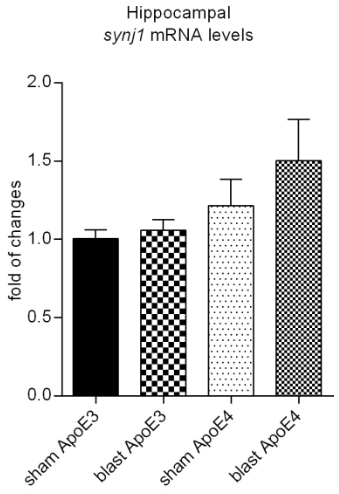

Figure 2 The *synj1* mRNA levels in hippocampal brain regions of ApoE3 and ApoE4 mice after blast TBI. Results are presented as fold changes relative to *synj1* mRNA levels in ApoE3 sham brains ±SEM. N=5/group, one-way ANOVA F(3,16)=1.871, p=0.175.

ApoE4-associated phospholipid dysregulation contributes to development of Tau hyper-phosphorylation after traumatic brain injury Jiqing Cao, Farida El Gaamouch, James S. Meabon, Kole D. Meeker, Li Zhu, Margaret B. Zhong, John Bendik, Gregory Elder, Ping Jing, Jiahong Xia, Wenjie Luo, David G. Cook, Dongming Cai

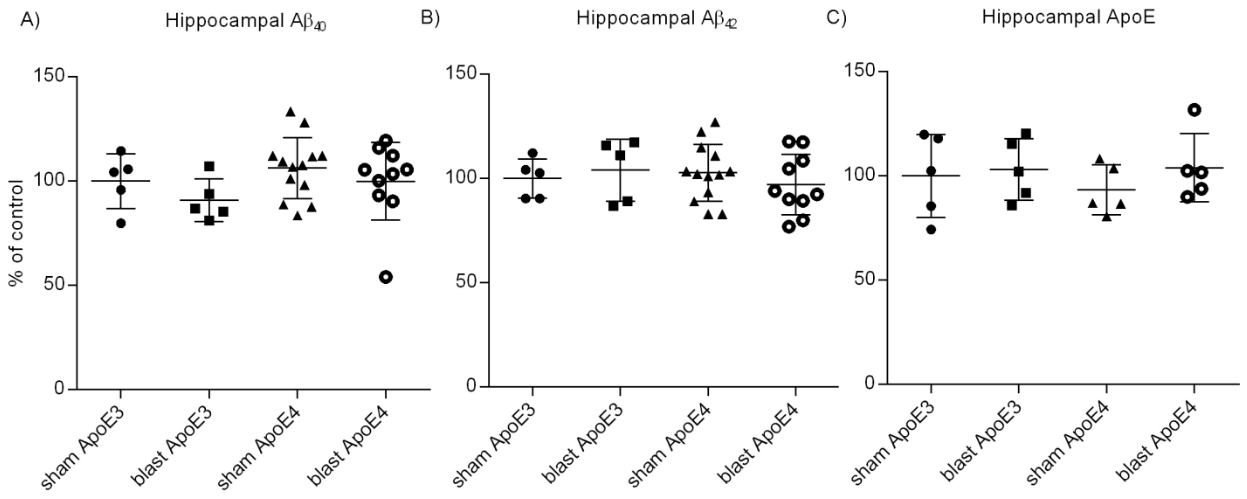

Figure 3 Levels of Aβ and ApoE in hippocampal brain regions of ApoE3 and ApoE4 mice in response to blast TBI. Amounts of A) mouse $A\beta_{40}$, B) mouse $A\beta_{42}$, and C) human ApoE in hippocampal regions of ApoE3 and ApoE4 mice were not significantly changed 24 hours post blast when compared to sham groups. Levels were calculated as pg/mg of total protein in brain lysates and presented as % of controls ±SEM (ApoE3 sham mouse brains). ApoE3 blast N=5, ApoE3 sham N=5, ApoE4 blast N=5-10 and ApoE4 sham N=5-13. A) one-way ANOVA $F(3,29)=1.260$, $p=0.3065$; B) one-way ANOVA $F(3,29)=0.4352$, $p=0.7294$; C) one-way ANOVA $F(3,16)=0.4545$, $p=0.7178$

ApoE4-associated phospholipid dysregulation contributes to development of Tau hyper-phosphorylation after traumatic brain injury Jiqing Cao, Farida El Gaamouch, James S. Meabon, Kole D. Meeker, Li Zhu, Margaret B. Zhong, John Bendik, Gregory Elder, Ping Jing, Jiahong Xia, Wenjie Luo, David G. Cook, Dongming Cai

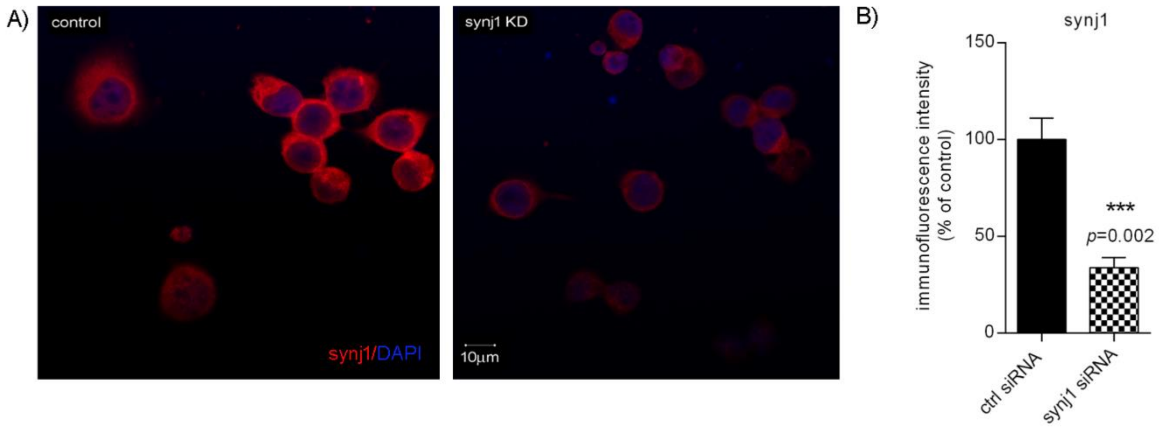

Figure 4 Levels of synj1 fluorescent signals in N2a cells. A) Immunofluorescence staining of synj1 (red) and DAPI (blue) in N2a cells of control versus synj1 KD conditions. B) Quantification of synj1 fluorescent signals indicate synj1 levels were reduced to 33.9% of controls in synj1 siRNA (KD) conditions (T(11.78)=5.385, *p*=0.002).

ApoE4-associated phospholipid dysregulation contributes to development of Tau hyper-phosphorylation after traumatic brain injury Jiqing Cao, Farida El Gaamouch, James S. Meabon, Kole D. Meeker, Li Zhu, Margaret B. Zhong, John Bendik, Gregory Elder, Ping Jing, Jiahong Xia, Wenjie Luo, David G. Cook, Dongming Cai

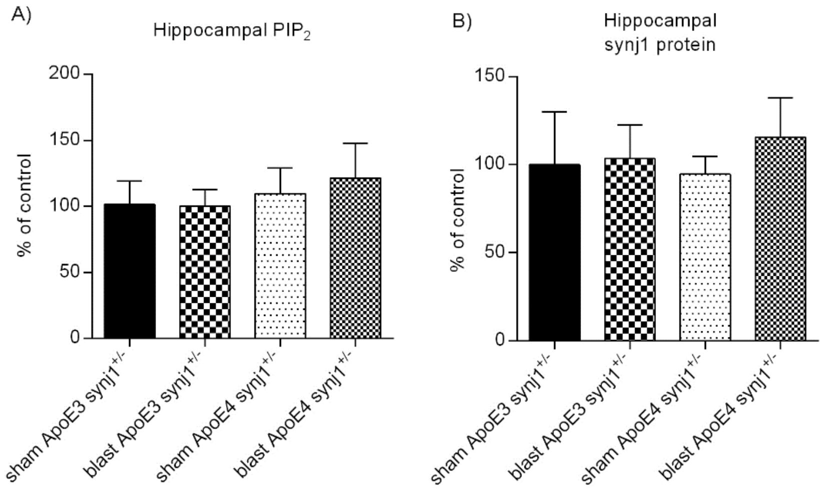

Figure 5 Levels of PIP_2 and synj1 in ApoE3 synj1$^{+/-}$ and ApoE4 synj1$^{+/-}$ mice in response to blast TBI. Levels A) PIP_2 and B) synj1 protein in hippocampal regions of ApoE3 synj1$^{+/-}$ and ApoE4 synj1$^{+/-}$ mice were not significantly changed 24 hours post blast when compared to sham groups. Results are presented as % of controls ±SEM (ApoE3 synj1$^{+/-}$ sham group). N=5/group. A) One-way ANOVA $F(3,16)=0.250$, $p=0.860$, B) one-way ANOVA $F(3,16)=0.168$, $p=0.917$.

ApoE4-associated phospholipid dysregulation contributes to development of Tau hyper-phosphorylation after traumatic brain injury Jiqing Cao, Farida El Gaamouch, James S. Meabon, Kole D. Meeker, Li Zhu, Margaret B. Zhong, John Bendik, Gregory Elder, Ping Jing, Jiahong Xia, Wenjie Luo, David G. Cook, Dongming Cai

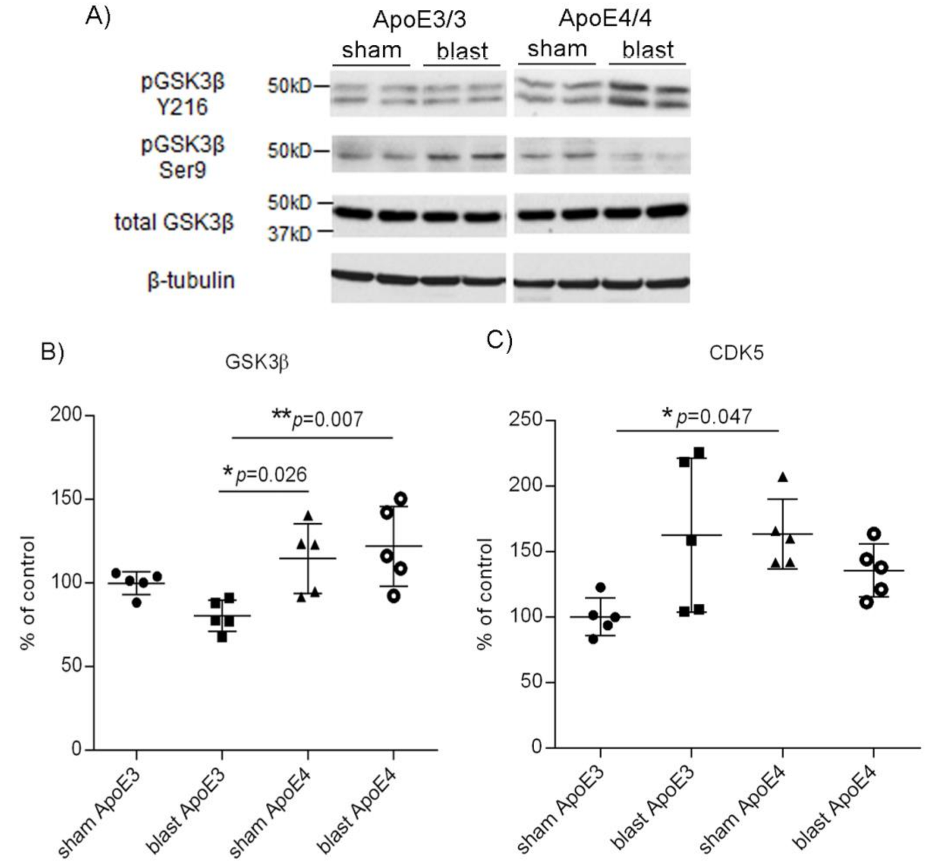

Figure 6 Levels of GSK3β and CDK5 in ApoE3 and ApoE4 mice in response to blast TBI. A) A representative example of western blot of active pGSK3β (Y216), inactive pGSK3β (Ser9) and total GSK3β in ApoE3 and ApoE4 mouse brains (sham versus blast). B) Levels of total GSK3β were higher in ApoE4 mouse brains (sham and blast) than those in ApoE3 counterparts. ApoE4 sham 114.8±20.9%, ApoE4 blast 122.0±24.0%, ApoE3 sham 100±6.8%, ApoE3 blast 80.5±9.4%; N=5/group, one-way ANOVA F(3,16)=5.883, *p*=0.0066; Tukey's multiple comparison test: ApoE4 blast *versus* ApoE3 blast *p*=0.007, ApoE4 sham *versus* ApoE3 blast *p*=0.026. C) Levels of CDK5 were not significantly changed in ApoE4 mice in response to blast exposure with a trend of increased CDK5 levels in ApoE3 mice after BOP exposure. The basal CDK5 levels in ApoE4 sham mice were higher than those of ApoE3 counterparts. ApoE4 sham

135.8±20.3%, ApoE4 blast 163.5±26.7%, ApoE3 sham 100.3±14.4%, ApoE3 blast 162.7±58.7%; N=5/group, one-way ANOVA F(3,16)=3.710, *p*=0.0336**.**

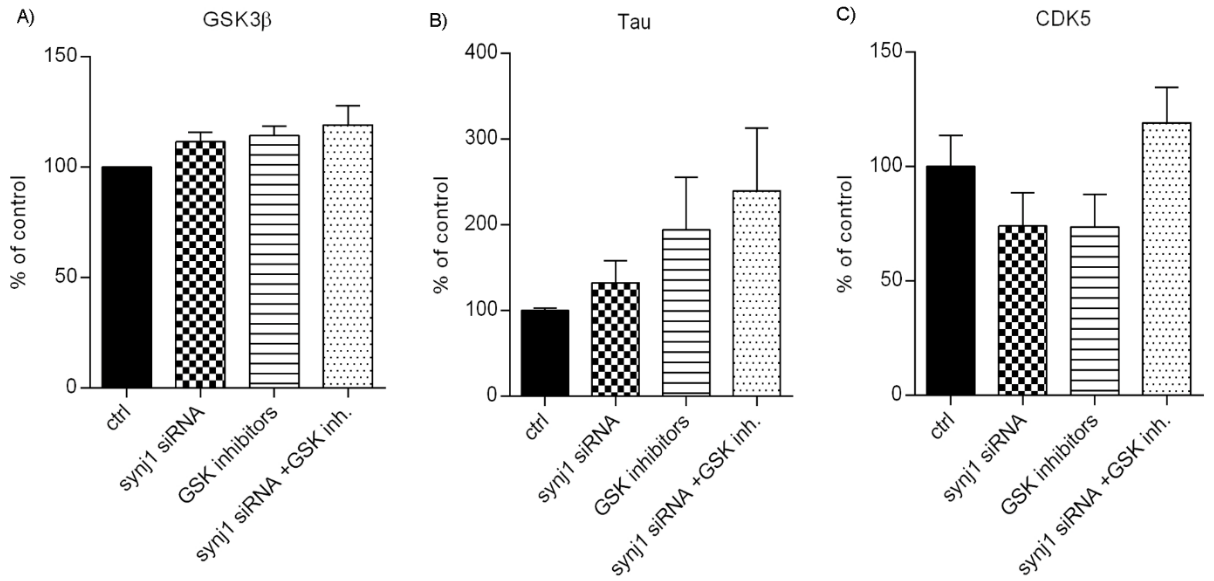

Figure 7 Levels of GSK3β, total Tau and CDK5 in N2a tau441 cells after treatments. No significant changes were seen in levels of A) total GSK3β, B) total Tau or C) CDK5 in any of these treatments (siRNA alone, GSK inhibitors, or combination of synj1 siRNA and GSK inhibitors). N=4-8/group. A) One-way ANOVA F(3,26)=1.183, *p*=0.336, B) one-way ANOVA F(3,19)=1.706, *p*=0.200, C) one-way ANOVA F(3,11)=2.283, *p*=0.136.
